# Treatment Options for Critically Ill Patients with Infections Caused by Metallo-Beta-Lactamase-Producing *Klebsiella pneumoniae*

**DOI:** 10.3390/antibiotics14111156

**Published:** 2025-11-14

**Authors:** Konstantinos Mantzarlis, Vassilios Vazgiourakis, Dimitrios Papadopoulos, Asimina Valsamaki, Stelios Xitsas, Masumi Tanaka, Achilleas Chovas, Efstratios Manoulakas

**Affiliations:** 1 Department of Critical Care, University Hospital of Larissa, School of Medicine, University of Thessaly, 41110 Larissa, Thessaly, Greece; vasvazg@yahoo.com (V.V.); semi_val@hotmail.com (A.V.); stratosfox@hotmail.com (E.M.); 2 Department of Critical Care, General Hospital of Larissa, 41221 Larissa, Thessaly, Greece; dcpapadopoulosmd@gmail.com (D.P.); achovas@yahoo.de (A.C.); 3 Department of Microbiology, University Hospital of Larissa, School of Medicine, University of Thessaly, 41110 Larissa, Thessaly, Greece; 4 Critical Care Department, King’s College Hospital NHS Foundation Trust, London SE5 9RS, UK

**Keywords:** MBL-producing *K. pneumoniae*, ceftazidime–avibactam + aztreonam, double carbapenem therapy, critically ill patients, survival

## Abstract

**Background/Objectives**: Antimicrobial resistance (AMR) has increased significantly over the years, contributing to a real challenge in the intensive care unit (ICU). The emergence of metallo-beta-lactamases (MBLs) has contributed to the protection of pathogens against all current beta-lactam/beta-lactamase inhibitors (BL/BLIs), including the newer ceftazidime–avibactam (CAZ-AVI), meropenem–vaborbactam, and imipenem–relebactam. Treatment of such infections is challenging. In vitro and clinical data suggest that combinations of CAZ-AVI with aztreonam (ATM) and the use of two different carbapenems (double carbapenem therapy, DCT) may be an option for MBL-producing pathogens. The aim of our study was to evaluate the effectiveness of the combination CAZ-AVI + ATM and the effectiveness of DCT against MBL-producing *K. pneumoniae* infections in the critically ill, mechanically ventilated patients. **Methods**: This is a retrospective study conducted in the two ICUs of hospitals in central Greece. Mechanically ventilated patients admitted to the ICU were included in the study if they developed an infection by MBL-producing *K. pneumoniae.* Patients were divided into three groups: the first one consisted of patients who were treated with CAZ-AVI plus ATM (CAZ-AVI + ATM group), and the second group consisted of patients who received DCT (DCT group). The third group included patients who received appropriate antibiotic therapy other than CAZ-AVI + ATM and DCT (control group). The primary outcome of the study was the evolution of the sequential organ failure assessment (SOFA) score, and secondary outcomes were duration of mechanical ventilation (MV), ICU length of stay (LOS), and, finally, ICU mortality. **Results**: 108 patients were included in the study. 35 (32%) in the CAZ-AVI + ATM group, 31 (29%) in the DCT group, and the remaining 42 (39%) patients in the control group. The SOFA score was not statistically different on day 1, day 4, and day 7 of the infection among the three groups (*p* > 0.05). Duration of MV and ICU LOS were also similar. Finally, mortality did not differ between the groups [20 patients (57.1%) vs. 18 (58.1%) vs. 25 (59.5%) for CAZ-AVI + ATM, DCT and control group, respectively, *p* = 0.98]. Comparison between survivors and non-survivors revealed that independent risk factors for mortality were SOFA score at day 1 of infection and medical cause of admission (*p* < 0.05). **Conclusions**: Treatment with CAZ-AVI + ATM or DCT presented similar efficacy with appropriate antibiotic therapy for infections caused by MBL-producing *K. pneumoniae* strains. Larger studies are required to confirm the findings.

## 1. Introduction

Antimicrobial resistance (AMR) is a leading cause of mortality, comorbidity and healthcare costs, contributing to a major concern for healthcare systems. It is estimated that in 2019 there were 4.95 million (95% UI 3.62–6.57) deaths associated with, and 1.27 million (0.911–1.71) deaths attributable to, multidrug-resistant bacteria [1]. According to a forecast for 2050, there will be 1.91 million (1.56–2.26) deaths attributable to, and 8.22 million (6.85–9.65) deaths associated with such infections. The total deaths attributable to AMR from 2025 to 2050 are estimated to be 39.1 million (33.0–46.0) [2]. AMR poses an important challenge for every clinician. Gram-negative bacterial (GNB) infections account for most AMR infections, especially in the intensive care unit (ICU) [3]. *Klebsiella pneumoniae* carbapenemase (KPC) has been the most prominent mechanism of resistance worldwide [4,5]. In recent years, new types of carbapenemases have emerged. Amongst them are the metallo-beta-lactamases (MBLs), carbapenemases, which protect pathogens from the new beta-lactamase inhibitors (BLIs) avibactam, vaborbactam, and relebactam [6], making the treatment of such infections challenging.

Two different strategies have been suggested for the treatment of such infections. The first one consists of the combination of ceftazidime–avibactam (CAZ-AVI) with aztreonam (ATM) [7,8]. AVI is active against carbapenemases other than MBLs. ATM cannot be hydrolyzed by MBLs, but it can be destroyed by carbapenemases other than MBLs. The combination of AVI and ATM is sufficient to counter pathogens’ mechanisms of resistance; AVI inhibits non-MBL carbapenemases, and ATM, which is not susceptible to MBLs, is then active against the pathogen. The second strategy is double-carbapenem therapy (DCT), and especially the combination of ertapenem and meropenem [9]. Ertapenem has a high affinity for carbapenemases and acts as a suicide inhibitor; in effect, carbapenemase activity decreases, and the second carbapenem can be active against the pathogen [10]. Clinical data for DCT is lacking, and thus, in the current guidelines, the combination of CAZ-AVI + ATM with cefiderocol is recommended against MBL-producing *Enterobacterales*; the combination of ATM and AVI is now also commercially available but was not available when the guidelines were released [11,12,13]. As such, we conducted a study to evaluate the effectiveness of the CAZ-AVI + ATM combination against DCT and other active antibiotics in the management of MBL-producing *K. pneumoniae* in the mechanically ventilated ICU patient population

## 2. Results

**Figure 1:**
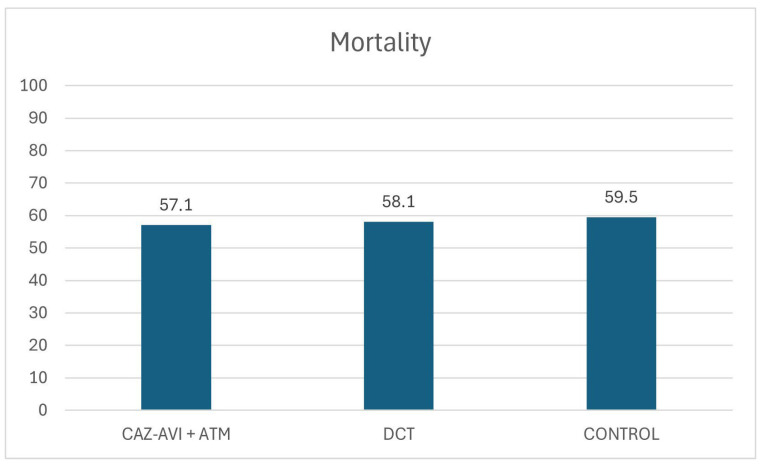
ICU mortality rates according to targeted antibiotic regimens. Abbreviations: CAZ-AVI, ceftazidime–avibactam; ATM, aztreonam; DCT, double carbapenem therapy.

A total of 108 patients presented with infection due to MBL-producing *K. pneumoniae*; 64 patients presented with bloodstream infection (BSI), and 44 patients presented with ventilator-associated pneumonia (VAP)—22 patients had been included in a previous study [14]. None of the patients was admitted to the ICU for the index infection. CAZ-AVI plus ATM was administered to 35 out of the 108 (32%) patients (CAZ-AVI + ATM group), DCT was administered in 31 (29%) patients (DCT group), and 42 (39%) patients received other effective antibiotic regimens (control group). Only the first episode was accounted. Twenty-three (65.7%) patients in the CAZ-AVI + ATM group suffered from BSI, and the remaining 12 (34.3%) from VAP. The DCT group (19; 61.3%) presented with BSI, and 12 (38.7%) with VAP; finally, 29 (69%) control group patients had BSI and 13 (31%) VAP. Twenty-one control group patients received as appropriate therapy gentamicin (50%), 14 patients received colistin (33%), 5 patients fosfomycin (12%), and the remaining 2 patients chloramphenicol (5%). Time to initiation of appropriate treatment was mean (SE) 8.4 (6.8) hours for CAZ-AVI + ATM group, 11 (5) for DCT group, and 19.2 (8.5) for control group (*p* = 0.59). Duration of therapy was [mean (SE) days] 11.8 (0.8) for CAZ-AVI + ATM group, 11.5 (1.6) for DCT, and 5.7 (1.6) for the control group (*p* = 0.057). The baseline characteristics of patients are presented in Table 1. There were statistically significant baseline differences between the three groups in age, sex, cause of ICU admission, days of prior antibiotic use, and liver disease in the medical history (*p* < 0.05). Patients presented with statistically similar mortality, ICU length of stay (LOS), mechanical ventilation (MV) duration, and sequential organ failure assessment (SOFA) scores on different days of infection. No differences were observed for inflammatory markers or SOFA scores for day 1 of infection (Table 2 and Table 3). There was no statistical difference in SOFA scores amongst days 1, 4 and 7 of infection (*p* > 0.05) (Table 3). Mortality was also similar between the groups (Figure 1) [20 patients died (57.1%) vs. 18 (58.1%) vs. 25 (59.5%) for CAZ-AVI + ATM, DCT and the control group, respectively, *p* = 0.98]. Also, there was no statistically significant difference in duration of mechanical ventilation (MV) [46.1 days (9.4) vs. 32.2 (3.8) vs. 30.9 (2.7) for CAZ-AVI + ATM, DCT and control group, respectively, *p* = 0.13] and ICU LOS [57.2 (12.4) vs. 39.6 (4.7) vs. 39.3 (3.6), *p* = 0.18] (Table 4). Comparison between survivors and non-survivors (Table 5) revealed that survivors were admitted in ICU more frequently for surgical reasons [20 (44.4%) survivors vs. 7 (11.1%) non-survivors, *p* = 0.0001], had lower SOFA score on day 1 of infection [5.1 (0.4) vs. 8.6 (0.5), *p* < 0.0001], higher markers of inflammation [c-reactive protein (CRP) 5.1 mg/dL (0.4) vs. 8.6 (0.5), *p* < 0.0001, and white blood cells (WBC) 10.8 × 10^9^/L (1.3) vs. 16.1 × 10^9^/L (1.3), *p* = 0.0011], and higher PaO_2_/FiO_2_ ratio [240 (4.5) vs. 154 (2.2), *p* < 0.0001]. After logistic regression, mortality was independently associated with surgical cause of admission [OR (95%CI), 0.144 (0.027–0785), *p* = 0.023] and SOFA score at day 1 of the index infection [1.536 (1.191–1.981), *p* = 0.001]. Only variables which were significantly associated with mortality after univariate analysis were included in the logistic regression model. None of the antibiotic regimens that the patients received independently affected mortality.

## 3. Discussion

In the present study, we aimed to evaluate the effectiveness of different antibiotic treatments for infections due to MBL-producing *K. pneumoniae.* CAZ-AVI + ATM and DCT were compared to appropriate antibiotic treatment. This is the first study, to our knowledge, that considers only critically ill, mechanically ventilated patients. The results indicate that CAZ-AVI + ATM presents with the same effectiveness as appropriate therapy or DCT.

Clinical data on the effectiveness of the combination therapy of CAZ-AVI + ATM for the treatment of MBL-producing pathogens is scarce. To date, the majority of the relevant literature consists of case reports or case series [15,16,17,18,19,20] with the exception of two prospective multi-centre observational studies by Falcone in 2021 and 2024 [21,22] and one interventional study [13]. The first of the observational studies [21] was conducted in Greece and Italy, where 102 patients with BSI were recruited. The 30-day mortality rate, which was the primary outcome, was 19.2% in the CAZ-AVI + ATM group vs. 44% in the best available treatment (BAT) group (*p* = 0.007). Secondary outcomes also favoured the CAZ-AVI + ATM combination. Propensity score adjusted analysis revealed lower clinical failure at day 14 [HR, 0.30 (95% CI, 0.14–0.65), *p* = 0.002], and shorter length of stay {HR, 0.49 (95% CI, 0.30–0.82), *p* = 0.007] CAZ-AVI + ATM [13]. The study concluded that the CAZ-AVI + ATM combination offered more favourable outcomes compared to BAT [13]. Our results do not confirm the above-mentioned results; however, differences in study populations may explain this discrepancy. In our study, the cohort consisted of mechanically ventilated ICU patients, while the majority of the patients in the study of Falcone were not ICU patients (30.4% of patients were mechanically ventilated), especially in the BAT group. Moreover, in the study of Falcone, 50% of the patients in the BAT group and 19.2% in the CAZ-AVI + ATM groups had a history of immunosuppressive treatment. Finally, the patients in our study suffered from both BSI and VAP. In the second observational study [22], the mortality was lower in the CAZ-AVI + ATM group (22.3%) compared to the group that received colistin-containing regimens (50%) or cefiderocol-containing regimens (33%), but higher than the group of patients who received other antibiotics (13.5%). In the same study, sensitivity analysis showed that CAZ-AVI + ATM was associated with reduced 30-day mortality rate [HR, 0.39, (95% CI, 0.18–0.86), *p* = 0.02] compared to colistin. Again, only 42% of the patients were admitted to the ICU. The REVISIT study [13] was a prospective, multi-centre study which recruited patients with complicated intra-abdominal infections or hospital-acquired pneumonia (including VAP); patients were randomized to ATM + AVI or meropenem with or without colistin. However, in REVISIT, there were seven patients with documented MBL-producing pathogens in the ATM + AVI group and three patients in the meropenem group, making a comparison with our study difficult.

There is insufficient clinical data about the efficacy of DCT for the treatment of MBL-producing *K. pneumoniae* infections. The existing studies are usually case reports and case series with a limited number of patients [23,24,25,26,27,28,29]. Clinical studies comparing DCT to other antibiotic combinations are also scarce. The first study [30] included patients with carbapenem-resistant *K. pneumoniae* who were treated with DCT. The control group consisted of patients who received standard therapy. The 28-day mortality was lower in the DCT compared to the control group (29.2% vs. 47.9%, respectively, *p* = 0.04). In the other two studies [31,32], DCT offered similar outcomes to standard therapy. On the other hand, a systematic review and meta-analysis [33] concluded that DCT presented a therapeutic advantage to other antibiotic therapies, as patients who received DCT had lower mortality [OR 0.44, 95% CI 0.24–0.82, (*p* = 0.009)]. Finally, two previous studies conducted by our group examining the impact of DCT in the ICU patients reported similar results [14,34]. These studies included ICU patients with infections caused by *K. pneumoniae* resistant to all available beta-lactam/b-lactamase inhibitors (BL/BLIs), and treatment with DCT presented similar outcomes to CAZ-AVI + ATM or other effective regimens. To our knowledge, this is the first study to consider DCT for MBL-producing *K. pneumoniae* infections in critically ill, mechanically ventilated patients.

The study presents several limitations. It was conducted in two hospitals in the same region, and the generalizability of the results may be limited. The number of patients was small. Hetero-resistance or other mechanisms of resistance were not studied. Sources of BSI are not specified, and moreover, side effects were not recorded. Finally, prior antibiotic treatments were not recorded, and thus, no effects on resistance mechanisms could be identified.

Overall, there is very little data available, and more high-quality clinical trials are required to address the efficacy and safety of the combination. Notwithstanding, CAZ-AVI + ATM or DCT could be considered as another therapeutic option.

## 4. Materials and Methods

This was a retrospective study between 2023 and 2025 conducted at the University Hospital of Larissa, Thessaly, Greece and the General Hospital of Larissa, Thessaly, Greece. Inclusion criteria were (a) admission to the ICU, (b) intubated and mechanically ventilated patients for >48 h, and (c) MBL-producing *K. pneumoniae* infection. Exclusion criteria were (a) age <18 years old, (b) ICU readmission, and (c) treatment with no effective antibiotics. If a patient presented with multiple episodes of infection, only the first one was accounted for. Patients were subsequently divided into three groups: patients treated with CAZ-AVI plus ATM (CAZ-AVI + ATM group); patients who received DCT (DCT group); and patients who received antibiotics other than CAZ-AVI + ATM and DCT, which were appropriate against the pathogen (control group). Patients in the first two groups presented infection due to pandrug-resistant (PDR) pathogens. On the other hand, control group patients had an infection secondary to extensively drug-resistant (XDR) *K. pneumoniae*, and they received appropriate therapy with the antibiotic to which the pathogen was sensitive.

### 4.1. Outcome

The primary outcome of the study was ICU mortality. Secondary outcomes were the evolution of the SOFA score during the course of infection, and more specifically on day 4 and day 7, duration of MV, and ICU LOS.

### 4.2. Definitions

The Centre for Disease Control (CDC) criteria were used for VAP (www.cdc.gov/nhsn/pdfs/pscmanual/10-vae_final.pdf, accessed on 10 May 2025) and BSI [35] definitions. As appropriate therapy was considered, the administration of in vitro active antibiotics for at least 48 h [36]. PDR is defined as a strain that is non-susceptible to all available antimicrobial agents, and XDR is a strain that is resistant to nearly all available antibiotics.

### 4.3. Antibiotics

CAZ-AVI and ATM were administered three times per day at doses of 2.5 g and 2 g, respectively. DCT consisted of ertapenem plus meropenem: 1 g of ertapenem was administered once a day before the first dose of meropenem; meropenem’s dose was 2 g every 8 h. All the above antibiotics were administered as extended infusions. All patients who received colistin or tigecycline received loading doses (9 million IU colistin and 200 mg tigecycline). Also, doses of all antibiotics were adjusted for creatinine clearance.

### 4.4. Clinical Assessment

The following patient characteristics were recorded: age, sex, medical history, surgical or medical admission, illness severity, duration of antibiotic treatment before ICU admission, and relevant clinical and laboratory findings for the index infection.

### 4.5. Microbiology

Identification and susceptibility testing of the isolated pathogens were performed by the Vitek 2 automated system (bioMérieux, Marcy-l’Étoile, Lyon, France). For the interpretation of the results, the European Committee on Antimicrobial Susceptibility Testing (EUCAST) breakpoints were used, and more specific v. 13.0 (2023), v. 14.0 (2023), and v 15.0 (2025). E-test was used to determine the minimum inhibitory concentration (MIC) for CAZ-AVI, meropenem−vaborbactam and imipenem−cilastatin−relebactam, and broth microdilution for colistin. Phenotypic methods were used for the detection of MBL-producing strains.

### 4.6. Statistical Analysis

Results are presented as frequencies (%) for categorical variables and as means (standard errors, SE) for continuous variables. Normality of data distribution was assessed by Kolmogorov/Smirnov test. Categorical variables were compared using the chi-square test or Fisher’s exact test, where appropriate; continuous variables were compared by the Mann-Whitney *U* test or the Kruskal–Wallis test. The Wilcoxon test was used to compare the variations in variables within the same group. Only variables with a *p*-value < 0.05 were used in the stepwise logistic regression models. GraphPad Prism 5 (GraphPad by Dotmatics Software Development, San Diego, CA, USA, 2007) software was used for data analysis.

## 5. Conclusions

The present study showed that ICU patients with infections caused by MBL-producing *K. pneumoniae* strains, who received CAZ-AVI + ATM or DCT, had similar clinical outcomes to those receiving other appropriate antibiotic regimens. The study was conducted in two hospitals in the same region with a relatively small number of patients. Mechanisms of resistance were not studied, and moreover, sources of BSI, prior antibiotic use, and side effects were not recorded. Therefore, larger studies are required to confirm these findings.

## Figures and Tables

**Table 1:** Baseline characteristics.

	CAZ-AVI + ATM (N = 35)	DCT (N = 31)	Control Group (N = 42)	All Patients (N = 108)	*p*-Value
Age (years)	56.7 (2.5)	62.3 (2.1)	64.1 (1.8)	61.3 (1.3)	0.037
Sex (male)	27 (77.1)	26 (83.9)	22 (52.4)	75 (69.4)	0.008
APACHE II score	16.8 (1.2)	16.2 (1.3)	14.9 (0.8)	15.9 (0.6)	0.413
SOFA score	7.5 (0.5)	7.5 (0.5)	7 (0.3)	7.3 (0.3)	0.627
Surgical cause of admission	9 (25.7)	3 (9.7)	15 (35.7)	27 (25)	0.04
Antibiotics prior to ICU (days)	9.2 (2.2)	2.2 (0.5)	3.4 (0.7)	4.9 (0.8)	<0.001
COPD	9 (25.7)	9 (29)	12 (28.6)	30 (27.8)	0.946
Heart Failure	8 (22.9)	2 (6.5)	4 (9.5)	14 (13)	0.098
Renal Failure	7 (20)	4 (12.9)	3 (7.1)	14 (13)	0.247
Liver Disease	4 (11.4)	0 (0)	0 (0)	4 (3.7)	0.013
Immunosuppression	1 (2.9)	4 (12.9)	1 (2.4)	6 (5.6)	0.106

Data is presented as a mean (SE) or n (%); CAZ-AVI, ceftazidime–avibactam; ATM, aztreonam; DCT, double carbapenem therapy; APACHE, acute physiology and chronic health evaluation; SOFA, sequential organ failure assessment; ICU, intensive care unit; COPD, chronic obstructive pulmonary disease; *p*, comparison between the three groups. Results after univariate analysis.

**Table 2:** Patients’ characteristics on day 1 of infection.

	CAZ-AVI + ATM (N = 35)	DCT (N = 31)	Control Group (N = 42)	All Patients (N = 108)	*p*-Value
WBC (10^9^/L)	14.8 (1.8)	13.2 (1.1)	13.7 (1.7)	13.9 (0.9)	0.802
Lymphocytes (10^9^/L)	1.6 (0.4)	1.3 (0.2)	1.2 (0.2)	1.3 (0.1)	0.633
CRP (mg/dL)	12 (1.6)	10.5 (1.8)	11.1 (1.4)	11.2 (0.9)	0.806
PaO_2_/FiO_2_ ratio	209 (19)	156 (12)	199 (9)	190 (10)	0.09
AKI (yes)	9 (25.7)	15 (48.4)	12 (28.6)	36 (33.3)	0.105

Data is presented as a mean (SE) or n (%); CAZ-AVI, ceftazidime–avibactam; ATM, aztreonam; DCT, double carbapenem therapy; WBC, white blood cell; CRP, C-reactive protein; AKI, acute kidney injury; *p*, comparison between the three groups. Results after univariate analysis.

**Table 3:** SOFA score for day 1, day 4, and day 7 of infection.

	CAZ-AVI + ATM (N = 35)	DCT (N = 31)	Control Group (N = 42)	*p*-Value
Day 1	7.2 (0.6)	7.5 (0.6)	6.8 (0.6)	0.699
Day 4	7.4 (0.7)	6.7 (0.7)	6 (0.6)	0.322
Day 7	7.1 (0.8)	5.9 (0.8)	5.7 (0.7)	0.374

Data is presented as mean (SE); CAZ-AVI, ceftazidime–avibactam; ATM, aztreonam; DCT, double carbapenem therapy; SOFA, sequential organ failure assessment; *p*, comparison between the three groups. Results by univariate analysis.

**Table 4:** Duration of MV, ICU stay, and mortality.

	CAZ-AVI + ATM (N = 35)	DCT (N = 31)	Control Group (N = 42)	*p*-Value
MV duration (days)	46.1 (9.4)	32.2 (3.8)	30.9 (2.7)	0.13
ICU LOS (days)	57.2 (12.4)	39.6 (4.7)	39.3 (3.6)	0.18
Mortality (%)	20 (57.1)	18 (58.1)	25 (59.5)	0.98

Data is presented as mean (SE) or n (%); CAZ-AVI, ceftazidime–avibactam; ATM, aztreonam; DCT, double carbapenem therapy; MV, mechanical ventilation; ICU, intensive care unit; LOS, length of stay; *p*, comparison between the three groups. Results by univariate analysis.

**Table 5:** Characteristics of survivors and non-survivors.

	Survivors (N = 45)	Non-Survivors (N = 63)	*p*-Value
Age (years)	59.7 (2)	62.4 (1.6)	0.324
Sex (male)	31 (68.9)	44 (69.8)	1
APACHE II score	15.3 (0.9)	16.3 (0.9)	0.338
Surgical cause of admission	20 (44.4)	7 (11.1)	<0.001
Antibiotics prior to ICU (days)	4.6 (1.5)	5.1 (0.9)	0.14
CAZ-AVI + ATM	15	20	1
DCT	13	18	1
COPD	9 (20)	21 (33.3)	0.191
Heart failure	7 (15.6)	7 (11.1)	0.567
Renal failure	5 (11.1)	9 (14.3)	0.774
Liver disease	1 (2.2)	3 (4.8)	0.639
Immunosuppression	4 (8.9)	2 (3.2)	0.232
Total ICU stay (days)	58 (9.5)	36 (3.2)	0.001
Total MV duration (days)	43.8 (7.4)	30.8 (2.5)	0.046
SOFA score (day of infection)	5.1 (0.4)	8.6 (0.5)	<0.001
WBC (10^9^/L) (day of infection)	10.8 (1.3)	16.1 (1.3)	0.001
Lymphocytes (10^9^/L) (day of infection)	1.2 (1.1)	1.5 (0.2)	0.402
CRP (mg/dl) (day of infection)	9.1 (1.5)	12.6 (1.1)	0.029
AKI (day of infection)	11 (24.4)	25 (39.7)	0.147
PaO_2_/FiO_2_ ratio (day of infection)	240 (14.5)	154 (12.2)	<0.001
Treatment duration (days)	14.9 (1.4)	12.1 (1)	0.122

Data are presented as mean (SE) or n (%); APACHE, acute physiology and chronic health evaluation; CAZ-AVI, ceftazidime–avibactam; ATM, aztreonam; DCT, double carbapenem therapy; COPD, chronic obstructive pulmonary disease; ICU, intensive care unit; MV, mechanical ventilation; SOFA, sequential organ failure assessment; WBC, white blood cells; CRP, C-reactive protein; AKI, acute kidney injury; *p*, comparison between the two groups. Results after univariate analysis.

## Data Availability

The original contributions presented in this study are included in the article. Further inquiries can be directed to the corresponding author.

## References

[1] Antimicrobial Resistance Collaborators (2022). Global burden of bacterial antimicrobial resistance in 2019: A systematic analysis. Lancet.

[2] GBD 2021 Antimicrobial Resistance Collaborators (2024). Global burden of bacterial antimicrobial resistance 1990-2021: A systematic analysis with forecasts to 2050. Lancet.

[3] Peleg A.Y., Hooper D.C. (2010). Hospital-acquired infections due to gram-negative bacteria. N. Engl. J. Med..

[4] Castanheira M., Deshpande L.M., Mendes R.E., Doyle T.B., Sader H.S. (2022). Prevalence of carbapenemase genes among carbapenem-nonsusceptible Enterobacterales collected in US hospitals in a five-year period and activity of ceftazidime/avibactam and comparator agents. JAC Antimicrob. Resist..

[5] Falcone M., Russo A., Iacovelli A., Restuccia G., Ceccarelli G., Giordano A., Farcomeni A., Morelli A., Venditti M. (2016). Predictors of outcome in ICU patients with septic shock caused by *Klebsiella pneumoniae* carbapenemase-producing *K. pneumoniae*. Clin. Microbiol. Infect..

[6] Mauri C., Maraolo A.E., Di Bella S., Luzzaro F., Principe L. (2021). The Revival of Aztreonam in Combination with Avibactam against Metallo-β-Lactamase-Producing Gram-Negatives: A Systematic Review of In Vitro Studies and Clinical Cases. Antibiotics.

[7] Sreenivasan P., Sharma B., Kaur S., Rana S., Biswal M., Ray P., Angrup A. (2022). In-vitro susceptibility testing methods for the combination of ceftazidime-avibactam with aztreonam in metallobeta-lactamase producing organisms: Role of combination drugs in antibiotic resistance era. J. Antibiot..

[8] Taha R., Kader O., Shawky S., Rezk S. (2023). Ceftazidime-Avibactam plus aztreonam synergistic combination tested against carbapenem-resistant Enterobacterales characterized phenotypically and genotypically: A glimmer of hope. Ann. Clin. Microbiol. Antimicrob..

[9] Giamarellou H., Galani L., Baziaka F., Karaiskos I. (2013). Effectiveness of a double-carbapenem regimen for infections in humans due to carbapenemase-producing pandrug-resistant *Klebsiella pneumoniae*. Antimicrob. Agents Chemother..

[10] Mashni O., Nazer L., Le J. (2019). Critical Review of Double-Carbapenem Therapy for the Treatment of Carbapenemase-Producing *Klebsiella pneumoniae*. Ann. Pharmacother..

[11] Paul M., Carrara E., Retamar P., Tängdén T., Bitterman R., Bonomo R.A., de Waele J., Daikos G.L., Akova M., Harbarth S. (2022). European Society of Clinical Microbiology and Infectious Diseases (ESCMID) guidelines for the treatment of infections caused by multidrug-resistant Gram-negative bacilli (endorsed by European society of intensive care medicine). Clin. Microbiol. Infect..

[12] Tamma P.D., Heil E.L., Justo J.A., Mathers A.J., Satlin M.J., Bonomo R.A. (2024). Infectious Diseases Society of America 2024 Guidance on the Treatment of Antimicrobial-Resistant Gram-Negative Infections. Clin. Infect. Dis..

[13] Carmeli Y., Cisneros J.M., Paul M., Daikos G.L., Wang M., Torre-Cisneros J., Singer G., Titov I., Gumenchuk I., Zhao Y. (2025). Aztreonam-avibactam versus meropenem for the treatment of serious infections caused by Gram-negative bacteria (REVISIT): A descriptive, multinational, open-label, phase 3, randomised trial. Lancet Infect. Dis..

[14] Mantzarlis K., Manoulakas E., Papadopoulos D., Katseli K., Makrygianni A., Leontopoulou V., Katsiafylloudis P., Xitsas S., Papamichalis P., Chovas A. (2025). Ceftazidime-Avibactam Plus Aztreonam for the Treatment of Blood Stream Infection Caused by *Klebsiella pneumoniae* Resistant to All Beta-Lactame/Beta-Lactamase Inhibitor Combinations. Antibiotics.

[15] Davido B., Fellous L., Lawrence C., Maxime V., Rottman M., Dinh A. (2017). Ceftazidime-Avibactam and Aztreonam, an Interesting Strategy To Overcome beta-Lactam Resistance Conferred by Metallo-beta-Lactamases in *Enterobacteriaceae* and *Pseudomonas aeruginosa*. Antimicrob. Agents Chemother..

[16] Shaw E., Rombauts A., Tubau F., Padullés A., Càmara J., Lozano T., Cobo-Sacristán S., Sabe N., Grau I., Rigo-Bonnin R. (2018). Clinical outcomes after combination treatment with ceftazidime/avibactam and aztreonam for NDM-1/OXA-48/CTX-M-15-producing *Klebsiella pneumoniae* infection. J. Antimicrob. Chemother..

[17] Hobson C.A., Bonacorsi S., Fahd M., Baruchel A., Cointe A., Poey N., Jacquier H., Doit C., Monjault A., Tenaillon O. (2019). Successful Treatment of Bacteremia Due to NDM-1-Producing *Morganella morganii* with Aztreonam and Ceftazidime-Avibactam Combination in a Pediatric Patient with Hematologic Malignancy. Antimicrob. Agents Chemother..

[18] Benchetrit L., Mathy V., Armand-Lefevre L., Bouadma L., Timsit J.F. (2020). Successful treatment of septic shock due to NDM-1-producing *Klebsiella pneumoniae* using ceftazidime/avibactam combined with aztreonam in solid organ transplant recipients: Report of two cases. Int. J. Antimicrob. Agents.

[19] Yasmin M., Fouts D.E., Jacobs M.R., Haydar H., Marshall S.H., White R., D’souza R., Lodise T.P., Rhoads D.D., Hujer A.M. (2020). Monitoring Ceftazidime-Avibactam and Aztreonam Concentrations in the Treatment of a Bloodstream Infection Caused by a Multidrug-Resistant Enterobacter sp. Carrying Both *Klebsiella pneumoniae* Carbapenemase-4 and New Delhi Metallo-beta-Lactamase-1. Clin. Infect. Dis..

[20] Shah P.J., Tran T., Emelogu F., Tariq F. (2021). Aztreonam, Ceftazidime/Avibactam, and Colistin Combination for the Management of Carbapenemase-Producing Klebsiella Pneumoniae Bacteremia: A Case Report. J. Pharm. Pract..

[21] Falcone M., Daikos G.L., Tiseo G., Bassoulis D., Giordano C., Galfo V., Leonildi A., Tagliaferri E., Barnini S., Sani S. (2021). Efficacy of Ceftazidime-avibactam Plus Aztreonam in Patients with Bloodstream Infections Caused by Metallo-beta-lactamase-Producing Enterobacterales. Clin. Infect. Dis..

[22] Falcone M., Giordano C., Leonildi A., Galfo V., Lepore A., Suardi L.R., Riccardi N., Barnini S., Tiseo G. (2024). Clinical Features and Outcomes of Infections Caused by Metallo-β-Lactamase-Producing Enterobacterales: A 3-Year Prospective Study From an Endemic Area. Clin. Infect. Dis..

[23] Ceccarelli G., Falcone M., Giordano A., Mezzatesta M.L., Caio C., Stefani S., Venditti M. (2013). Successful ertapenem-doripenem combination treatment of bacteremic ventilator-associated pneumonia due to colistin-resistant KPC-producing *Klebsiella pneumoniae*. Antimicrob. Agents Chemother..

[24] Oliva A., D’Abramo A., D’Agostino C., Iannetta M., Mascellino M.T., Gallinelli C., Mastroianni C.M., Vullo V. (2014). Synergistic activity and effectiveness of a double-carbapenem regimen in pandrug-resistant *Klebsiella pneumoniae* bloodstream infections. J. Antimicrob. Chemother..

[25] Oliva A., Mascellino M.T., Cipolla A., D’aBramo A., De Rosa A., Savinelli S., Ciardi M.R., Mastroianni C.M., Vullo V. (2015). Therapeutic strategy for pandrug-resistant *Klebsiella pneumoniae* severe infections: Short-course treatment with colistin increases the in vivo and in vitro activity of double carbapenem regimen. Int. J. Infect. Dis..

[26] Chua N.G., Zhou Y.P., Tan T.T., Lingegowda P.B., Lee W., Lim T.P., Teo J., Cai Y., Kwa A.L. (2015). Polymyxin B with dual carbapenem combination therapy against carbapenemase-producing *Klebsiella pneumoniae*. J. Infect..

[27] Camargo J.F., Simkins J., Beduschi T., Tekin A., Aragon L., Perez-Cardona A., Prado C.E., Morris M.I., Abbo L.M., Cantón R. (2015). Successful Treatment of Carbapenemase-Producing Pandrug-Resistant *Klebsiella pneumoniae* Bacteremia. Antimicrob. Agents Chemother..

[28] El Nekidy W.S., Mooty M.Y., Attallah N., Cardona L., Bonilla M.F., Ghazi I.M. (2017). Successful treatment of multidrug resistant *Klebsiella pneumoniae* using dual carbapenem regimen in immunocompromised patient. IDCases.

[29] Piedra-Carrasco N., Miguel L., Fàbrega A., Viñado B., Campany D., Mir A., Fox M.L., Almirante B., Larrosa N., Ruiz-Camps I. (2018). Effectiveness of a Double-Carbapenem Regimen in a KPC-Producing *Klebsiella pneumoniae* Infection in an Immunocompromised Patient. Microb. Drug Resist..

[30] De Pascale G., Martucci G., Montini L., Panarello G., Cutuli S.L., Di Carlo D., Di Gravio V., Di Stefano R., Capitanio G., Vallecoccia M.S. (2017). Double carbapenem as a rescue strategy for the treatment of severe carbapenemase-producing *Klebsiella pneumoniae* infections: A two-center, matched case-control study. Crit. Care.

[31] Venugopalan V., Nogid B., Le T.N., Rahman S.M., Bias T.E. (2017). Double carbapenem therapy (DCT) for bacteremia due to carbapenem-resistant *Klebsiella pneumoniae* (CRKP): From test tube to clinical practice. Infect. Dis..

[32] Oliva A., Scorzolini L., Castaldi D., Gizzi F., De Angelis M., Storto M., D’ABramo A., Aloj F., Mascellino M., Mastroianni C. (2017). Double-carbapenem regimen, alone or in combination with colistin, in the treatment of infections caused by carbapenem-resistant *Klebsiella pneumoniae* (CR-Kp). J. Infect..

[33] Li Y.Y., Wang J., Wang R., Cai Y. (2020). Double-carbapenem therapy in the treatment of multidrug resistant Gram-negative bacterial infections: A systematic review and meta-analysis. BMC Infect. Dis..

[34] Mantzarlis K., Manoulakas E., Parisi K., Sdroulia E., Zapaniotis N., Tsolaki V., Zakynthinos E., Makris D. (2024). Meropenem plus Ertapenem and Ceftazidime-Avibactam plus Aztreonam for the Treatment of Ventilator Associated Pneumonia Caused by Pan-Drug Resistant Klebsiella pneumonia. Antibiotics.

[35] Garner J.S., Jarvis W.R., Emori T.G., Horan T.C., Hughes J.M. (1988). CDC definitions for nosocomial infections. Am. J. Infect. Control.

[36] Zarkotou O., Pournaras S., Tselioti P., Dragoumanos V., Pitiriga V., Ranellou K., Prekates A., Themeli-Digalaki K., Tsakris A. (2011). Predictors of mortality in patients with bloodstream infections caused by KPC-producing *Klebsiella pneumoniae* and impact of appropriate antimicrobial treatment. Clin. Microbiol. Infect..

